# Aspects of Facial Contrast Decrease with Age and Are Cues for Age Perception

**DOI:** 10.1371/journal.pone.0057985

**Published:** 2013-03-06

**Authors:** Aurélie Porcheron, Emmanuelle Mauger, Richard Russell

**Affiliations:** 1 CE.R.I.E.S. – the CHANEL R&T skin research center on healthy skin, Neuilly-sur-Seine, France; 2 Gettysburg College, Gettysburg, Pennsylvania, United States of America; University of Leuven, Belgium

## Abstract

Age is a primary social dimension. We behave differently toward people as a function of how old we perceive them to be. Age perception relies on cues that are correlated with age, such as wrinkles. Here we report that aspects of facial contrast–the contrast between facial features and the surrounding skin–decreased with age in a large sample of adult Caucasian females. These same aspects of facial contrast were also significantly correlated with the perceived age of the faces. Individual faces were perceived as younger when these aspects of facial contrast were artificially increased, but older when these aspects of facial contrast were artificially decreased. These findings show that facial contrast plays a role in age perception, and that faces with greater facial contrast look younger. Because facial contrast is increased by typical cosmetics use, we infer that cosmetics function in part by making the face appear younger.

## Introduction

Age is a fundamental determinant of social structure and interactions. Age determines rank, rights, and responsibilities. People of different ages afford different kinds of social interactions and age is a primary dimension of social cognition and behavior. But it is not only *actual* or *chronological* age that predicts a person’s health, ability, and treatment by others. The mere *appearance* of age, separate from actual age, predicts important aspects of health and well-being.

Looking older or younger than one’s age is associated with health and environmental factors such as body mass index (BMI), depression, marital status, and social class [1], [2]. Indeed, perceived facial age is a clinically useful biomarker of aging [3], and looking older than one’s age is a sign of poor health [4], [5] and mortality [6]. Though poor health surely contributes to appearing old for one’s age, there is evidence to suggest that appearance may also *cause* diminished health and psychological well-being, because of reduced social contact and social touching that results from having skin that no one “loves to touch”, including the possessor [7]. A person who appears older is perceived as more autonomous and dominant [8], which discourages touching [9]. Though the benefits of touch increase with age, the opportunities to be touched decrease significantly [10].

Maintaining a youthful appearance is of great importance for many people, perhaps because of the relationships between the appearance of age and health, and between the appearance of age and beauty [11]. Many people are concerned with reducing the visual signs of aging, and this supports the existence of the multi-billion dollar cosmetic and cosmetic surgery industries.

The appearance of age is closely related to the physical changes that occur with the aging process. After the cessation of growth at approximately 20 years of age, face shape continues to change, particularly in late adulthood [12]. Facial skin undergoes dramatic changes with age, including wrinkling and sagging [13]–[16], increases of pigmented irregularities, and skin color changes such as decreased homogeneity of skin reflectance [17], [18].

The internal features of the face are also relevant to the perception of age. With photographs of the same individual obtained at two different ages, George and Hole substituted features between the photographs. Transplanting older features into a younger face increased age estimates by approximately 40%, the opposite decreased the age of the older face by approximately 33% [19]. Both internal feature size and shape influence age perception. Large and round eyes in real faces as well as shorter noses decreased the estimated age of the person [8]. Lip height and border definition decrease with age and are visual cues for age perception [20], [21].

The luminance contrast between the eyes and the surrounding skin and the lips and the surrounding skin has been termed ‘facial contrast’. Female faces have greater facial contrast than male faces, and facial contrast plays an important role in sex classification and the perception of masculinity and femininity and also attractiveness [22]–[26]. However, it is not known whether facial contrast changes with age or plays a role in age perception. Inspection of averaged faces of older and younger adults led us to hypothesize that facial contrast decreases with age and is related to perceived facial age.

We are extending the definition of facial contrast to include the eyebrows because they are a major source of perceptually relevant contrast in the face [27]. Eyebrow contrast may be specifically important for age perception, since facial hair becomes gray and of lesser quantity with age. Elsewhere we have focused on luminance contrast only [24], [25], but recent work has demonstrated the importance of color contrast between the features and the surrounding skin for sex classification and related face perception tasks [22], [23], [26]. For this reason we investigated not only age-related changes in luminance contrast, but also in red – green and yellow – blue contrast.

In the first study, we measured facial contrast in a set of 289 facial images of Caucasian women aged between 20 and 70 years old, to determine whether facial contrast varies with age. To determine whether facial contrast is related to age perception we had participants estimate the age of 150 of the faces in a second study. In the third study we manipulated facial contrast to determine whether it is causally related to perceived age. Each of thirty faces was manipulated to create new versions of the face with either increased or decreased facial contrast. In a forced-choice design, participants were shown both modified images and instructed to determine which face looked younger. A second group of participants were shown the modified versions of each face individually and asked to estimate its age.

## Study 1

We conducted study 1 to test the hypothesis that luminance and color contrast of women faces changes with age.

### Ethics Statement

We have obtained ethics approval for our study from the Gettysburg College Institutional Review Board for Research with Human Subjects and the research followed the principles of the Helsinki Convention. The subjects reported in this manuscript have given written informed consent. The individual pictured in this manuscript has given written informed consent for her images (altered as well as unaltered versions) to appear in a scientific publication, with the understanding that her name and/or personal information will not be made public.

### Materials and Procedure

Full face images of 289 French Caucasian women with healthy skin between 20 to 69 years old (40 faces from 20 to 29 years, 60 faces from 30 to 39 years, 71 faces from 40 to 49 years, 60 faces from 50 to 59 years, and 58 faces from 60 to 69 years) were acquired using a closed photographic system that allows accurate and reproducible positioning of the subjects as well as controlled lighting conditions. Written informed consent was obtained from all subjects allowing the use of their photographs for research studies.

The height of the camera (Canon EOS-1 Ds Mark II, 17 MP) was adjusted to the height of the face. Each face was illuminated by three flashes: one in front of the face (diffuse light), the height of this flash was adjusted to the height of the subject’s face; and two flashes illuminating the face from a 45 angle (direct light), the height of these flashes was fixed. These lighting conditions were defined in order to avoid cast shadows and to minimize variation from shading on the faces. The subjects wore no make-up or adornments. The vast majority of the subjects (263 out of 289 total) wore a headband to keep their hair away from their face. Subjects’ eyes were open, and they were asked to keep a neutral expression and gaze directly into the camera. Faces wearing permanent make-up or colored contact lenses were not included. The images were cropped to leave the face contour visible.

Because this was a cross-sectional study, we wanted to determine whether any changes of eye contrast with age could be due to differences in iris color between the young and the older women of our study. Toward this end, the iris color of each face was evaluated using the system described by Seddon et al. [28] We analyzed the difference in eye color between the older and younger faces in our set of images with a χ^2^ test, and found no significant difference in iris color between the different ten-year age classes (*p* = 0.65).

**Figure 1:**
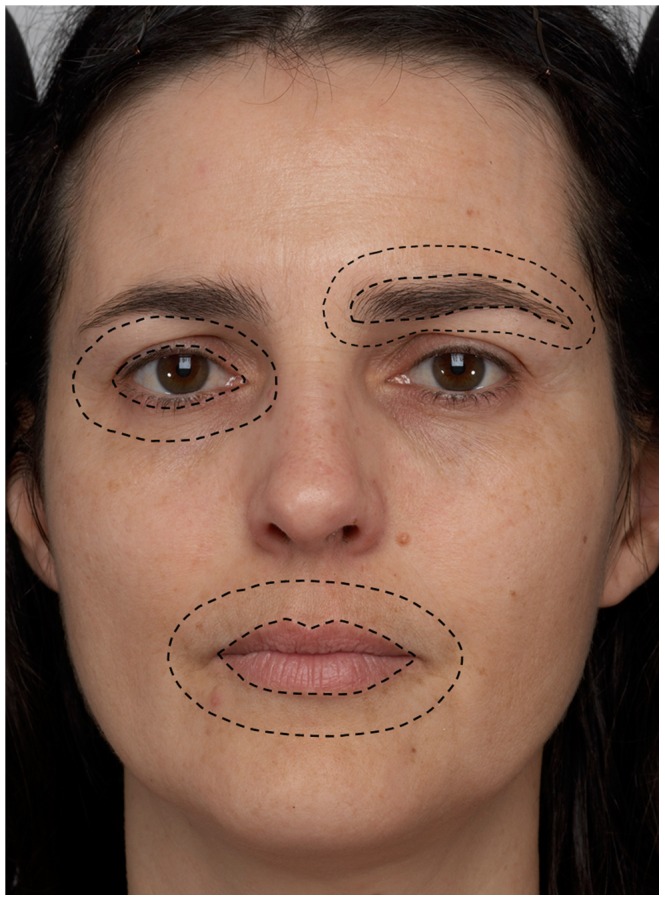
Labelling of facial regions. The dashed lines demonstrate how the features and surrounding skin were defined.

The labeling of facial regions and the measurement of the contrast was performed using MATLAB 7.8.0 (R2010a). Similar to the procedures of Russell, 2009 [25], each image was individually labeled to define regions corresponding to the eyes (including a narrow band of skin around the lashes), the lips, the eyebrows, annuli surrounding the eyes (with the approximate width of the eyes but not including the eyebrows), an annulus surrounding the lips (with the approximate width of the mouth), annuli surrounding the eyebrows (with the approximate width of the eyebrows) and the face contour (**Figure 1**).

To measure the luminance and color contrast of the face we used the CIE L*a*b* color space which corresponds roughly to the color channels of the human visual system. L*a*b* color space was designed such that differences between coordinates of stimuli are predictive of perceived color difference between the stimuli [29]. The three orthogonal dimensions of this color space are light-dark (L*), red-green (a*), and yellow-blue (b*).

Luminance values of all pixels within the eyes were averaged, as were all the pixels in the other features and the annuli surrounding the features. Skin and feature luminance, both being the averages of 8-bit pixel values, could range from 0 (black) to 255 (white). The contrast was calculated for each feature as C_f_ = (skin luminance – feature luminance)/(skin luminance+feature luminance). This is an adapted version of Michelson contrast, which varies from −1 to 1, with higher absolute values indicating greater contrast, and 0 indicating no contrast. The same method was applied to measure red-green and yellow-blue facial contrast, with a* ranging from 0 (green) to 255 (red) and b* ranging from 0 (blue) to 255 (red). A descriptive analysis of the facial contrast was performed and the relationships between age and contrasts were tested using a Pearson correlation.

### Results

The faces had positive luminance contrast for the eyes, lips, and brows, indicating that in all faces the eyes, lips, and brows were darker than the surrounding skin. The a* contrast of the mouth was found to be negative in all the faces, because the mouth is redder than the surrounding skin (for further analysis this indicator will be treated in absolute values). The b* contrast of the mouth as well as the a* and b* contrast of the eyes was positive in nearly all the faces, indicating that in most faces the mouth is more blue than the surrounding skin, while the eyes are more blue and green than the surrounding skin. The mean color contrast (a* and b*) of the eyebrows was not significantly different from zero, and these contrasts were removed from the subsequent statistical analysis.

**Figure 2:**
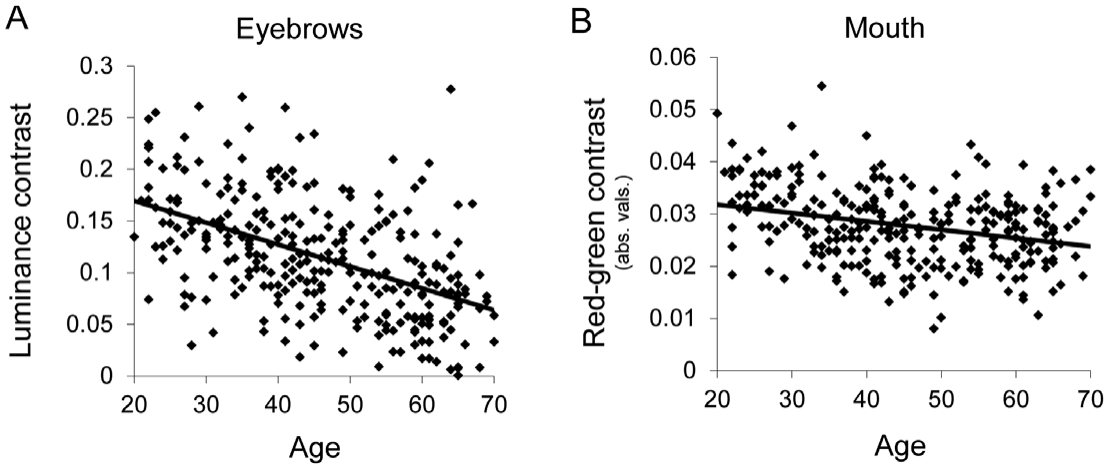
Figure 2. Decrease in contrast with age. L* contrast of the eyebrows (left) and a* contrast of the mouth (right) as a function of age of the face. Each point represents a particular face.

The relationship between age and luminance and color contrast is presented in **Table 1**, while **Figure 2** shows graphs of two of the contrasts (luminance contrast around the brow and a* contrast around the mouth). There were several changes in facial contrast with age. We observed a significant and strong decrease of luminance contrast of the eyebrow region with age (p<0.0001), whereas only a trend to decrease with age was found for the eye region (p = 0.06). The a* contrast (absolute value) significantly decreased with age for the mouth region (p<0.0001) and the eye region (p<0.0001). The b* contrast significantly decreased with age for the eye region (p<0.0001) but increased for the mouth region (p<0.01). The luminance contrast of the mouth region was unrelated to the age of the face. Collectively, these findings show that aspects of facial contrast change with age, with most of these components decreasing with age.

**Table 1:** The relationship between age and luminance (L*) or color (a*, b*) contrast.

Feature	Contrast	N	*p* value	Correlation coeff.
Brows	L*	289	<0.0001	−0.50
Eyes	L*	289	0.0645	−0.11
Mouth	L*	289	0.6625	0.03
Eyes	a*	289	<0.0001	−0.32
Mouth^1^	a*	289	<0.0001	−0.29
Eyes	b*	289	<0.0001	−0.24
Mouth	b*	289	0.0081	0.16

1 *Absolute value.*

## Study 2

Having found that aspects of facial contrast decrease with age, we wished to determine whether facial contrast plays a role in the perception of age. Because so many cues other than facial contrast are known to play a role in age perception (as reviewed in the Introduction), we do not expect facial contrast to perfectly predict perceived age, but nonetheless to be significantly correlated with perceived age. Study 2 tested the hypothesis that there is a relationship between facial contrast and the perceived age of the face.

### Materials and Procedure

For Study 2 we selected 150 of the images from Study 1 that were homogeneously distributed according to the age of the women and the color of their iris. Seventy-four Gettysburg College undergraduates (17–22 years, 36 males and 38 females) participated in Experiment 2. All 150 faces were presented to each participant, one at a time in random order. The participants were asked to estimate the age of each face, between 10 and 99 years with a keyboard response and no time limit.

A descriptive analysis of perceived age was performed, and the relationships between perceived age and the different components of facial contrast were tested using analysis of variance with mixed effects. The term mixed model refers to the use of both fixed and random effects in the same analysis. The mixed model methodology is generally used when a variable is measured for the same individual repeatedly over time or under various experimental conditions (in our study the faces’ estimated age). In our model, the different aspects of facial contrast, participants age and participant gender are fixed effects, while participant identity and face identity are random effects. For further description of mixed models, see Verbeke and Molenberghs [30], and for use of mixed models in similar research, see Stephen and McKeegan [26].

### Results

On average, the participants perceived the faces as slightly older than they actually were, with a mean age difference (perceived age – actual age) of 2.8 years (Standard Deviation = 8.7). We found no significant effect of participant age or gender on perceived age. These relationships are shown in **Table 2**. The components of facial contrast were negatively correlated with estimated age, except for b* contrast of the mouth which was positively correlated with perceived age, and the luminance contrast of the mouth which was not correlated with perceived age. Luminance contrast of the eyebrow (p<0.0001) and a* contrast of the mouth (p<0.0001) were the factors most strongly associated with the estimated age of the faces, followed by the a* contrast of the eye region (p<0.001), and the b* contrast of the mouth region (p<0.01) and eye region (p = 0.02). There was a trend toward luminance contrast of the eye region predicting the age of the faces (p = 0.05). In general, faces with greater contrast were perceived as younger. Specifically, only those aspects of facial contrast that change with age were found to be linked with perceived age.

**Table 2:** The relationship between perceived age and luminance (L*) or color (a*, b*) contrast.

Feature	Contrast	N	*p* value	Relationship to perceived age
Brows	L*	150	<0.0001	Decrease
Eyes	L*	150	0.0529	Decrease
Mouth	L*	150	0.4513	None
Eyes	a*	150	0.0009	Decrease
Mouth 1	a*	150	<0.0001	Decrease
Eyes	b*	150	0.0160	Decrease
Mouth	b*	150	0.0069	Increase

1 *Absolute value.*

## Studies 3a and 3b

In the third study we manipulated those aspects of facial contrast that vary with age to determine whether they are causally related to age perception. Two versions of the experiment were run; one with a forced-choice design in which the two versions of each face were compared directly (Study 3a) and the second with participants estimating the age of the faces one at a time (Study 3b). The two versions are both ecologically valid tasks insofar as it is common to make a comparative estimate of the age of two (or more) different people (i.e. who is younger) as well as to make an estimate of a single individual’s age (i.e. approximately how old is this person). The forced-choice design is the most sensitive way to determine whether facial contrast plays a role in age perception, because participants directly compare two versions of the same face that differ only in terms of the manipulated aspects of facial contrast. By also running the ratings design we were able to replicate the results with a different design, and also to measure the size of the effect of the manipulation and to compare it with other studies that have used similar designs.

### Materials

Thirty images were selected from the set of 150 used in Experiment 2 in order to have 10 faces of women 23 to 34 years old, 10 faces of women 35 to 44 years, and 10 faces of women 45 to 59 years.

Each face was manipulated to increase or decrease only those aspects of facial contrast that were observed to be significantly lower with age, or for which there was a trend toward being lower with age. Specifically, we manipulated the L* contrast around the eyebrows and eyes, the a* contrast around the eyes and lips, and the b* contrast around the eyes and lips. L* contrast around the mouth was not manipulated because this had not been found to vary with age. To manipulate contrast around a feature, the features were manipulated while the surrounding skin was left unchanged (i.e., the luminance of the eyes and the eyebrows, the redness of the eyes and lips, and the yellowness of the eyes and lips were increased or decreased). The burn tool in Adobe Photoshop® was used to selectively darken the eyebrows and the dodge tool was used to selectively lighten the eyebrows. To manipulate the size of the L*, a*, and b* contrast between the eyes and the surrounding skin and the a*, and b* contrast between the mouth and the surrounding skin, we individually manipulated the L*, a* or b* channel (0 to 255) of the relevant feature without changing the rest of the face. For instance, increasing the a* value of the lips made the lips redder and led to an increase of the a* contrast between the lips and the skin surrounding the lips.

**Figure 3:**
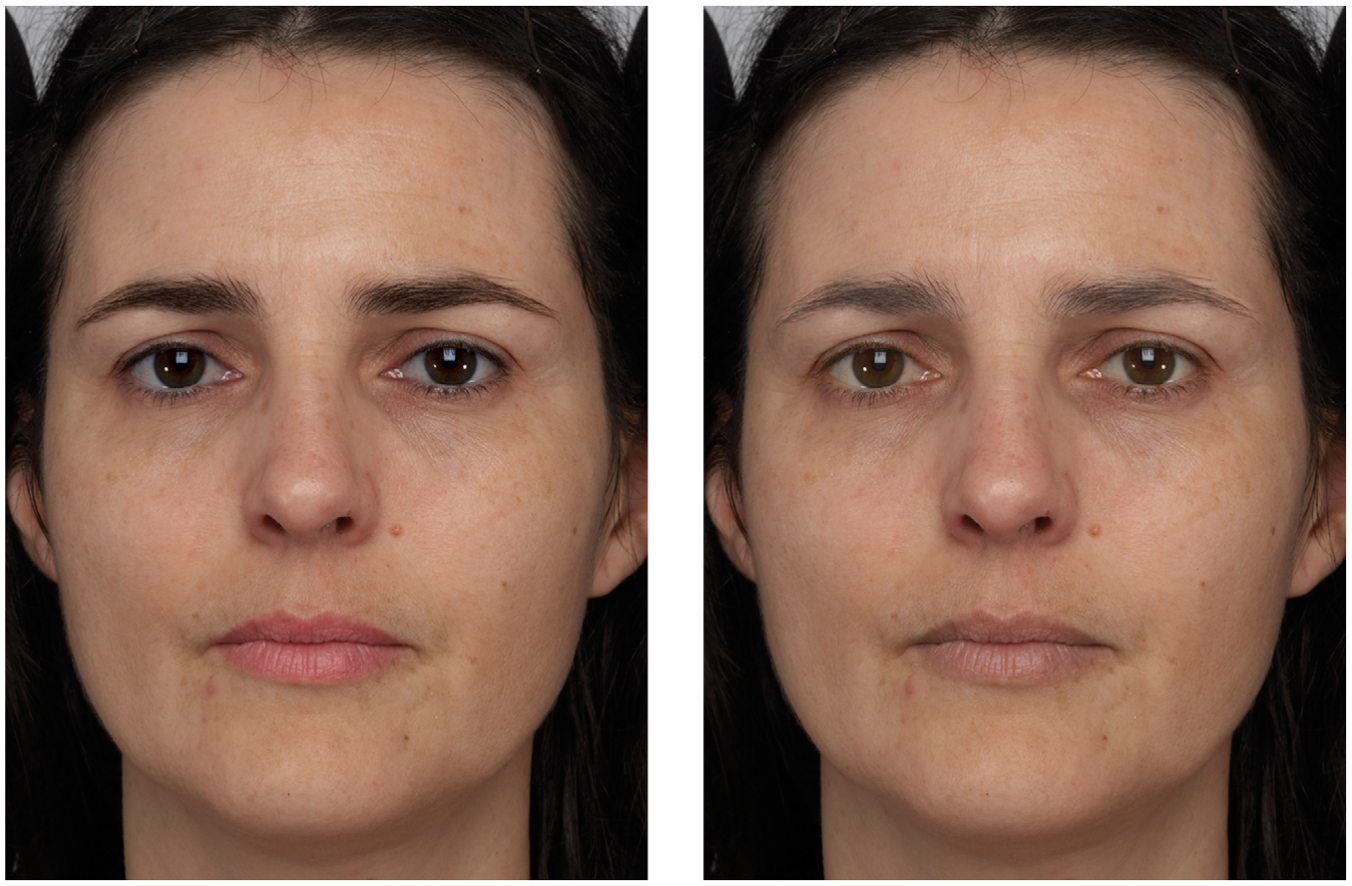
Contrast manipulated versions of a face. The left image shows a face with facial contrast increased and the right image shows the same face with facial contrast decreased.

For the present study our goal was to determine whether these aspects of facial contrast played any role in age perception and so we selected for each face the magnitude of change for each feature that seemed to maximize the change to apparent age while maintaining a naturalistic appearance. In practice, the magnitude of the manipulation was the same for most faces, but was made weaker in some faces in order to maintain a naturalistic appearance. The features were defined as described in Study 1. Only the manipulated faces (low/high contrast) were presented to the participants. Example stimuli are shown in **Figure 3**.

It is worth noting several things from Figure 3. The first is that the face with higher contrast (on the left) quite clearly appears younger than the face with lower contrast (on the right), as do other pairs of faces created in this way. This observation foreshadows the major results of Studies 3a and 3b. Several people who have seen these images have also observed that the entire face appears different, not just those parts of the face that were manipulated. For example, the skin of the face on the right appears somewhat less saturated (‘drained of color’), and generally less healthy than the face on the left. We presume that the changed appearance of regions of the face that were not manipulated is due to the holistic nature of face processing in general, and to the holistic nature of age perception [31] in specific. This also suggests that, as well as looking younger, the face with higher contrast appears healthier and more attractive than the face with lower contrast, which may be related to recent findings that facial color affects perceived health [32], [33].

### Study 3a: Procedure and Results

Twenty-one Gettysburg College undergraduates (10 females and 11 males, 18 to 20 years old) participated in study 3a. For each of the 30 stimulus faces, participants saw both the contrast-increased and contrast-decreased versions presented side-by-side and indicated with a button press which looked younger. The sequence of identities was counterbalanced for age and randomized for each participant, and the left-right ordering of high/low contrast versions were counterbalanced.

**Figure 4:**
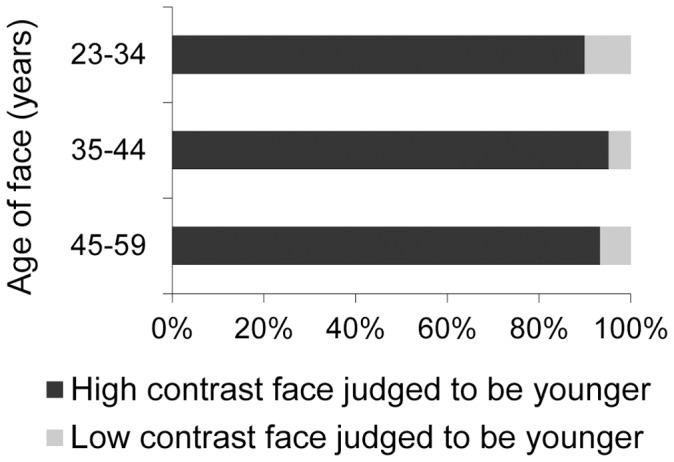
Results of forced-choice contrast manipulation experiment. The percentage of trials for which the high contrast face was judged younger is shown in the dark bars and the percentage of trials for which the low contrast face was judged younger is shown in the light bars. Results are shown for three age classes.

In 93% of trials the participants indicated that the high contrast face looked younger. This overwhelming effect indicates that participants used facial contrast as a cue in perceiving age, and that faces with increased facial contrast were perceived as younger. In order to determine whether the effect size was different for faces of different ages, we used a logistic regression model with repeated measurements. We found no interaction between the effect of the facial contrast manipulation and the age of the face. This means that the effect of facial contrast was not different for faces of different ages. The results from study 3a are shown in **Figure 4**.

### Study 3b: Procedure and Results

Eighty-one participants (60 females and 21 males) participated in experiment 3b at the CE.R.I.E.S. laboratory in Paris. Of the 60 females, 20 were between 18 and 22 years, 20 were between 37 and 42 years, and 20 were between 53 and 57 years. The 21 men were between 37 and 42 years. Therefore the effect of the age of the participants was tested by comparing the three groups of female participants, and the effect of the gender of the participants was tested by comparing the men with the women aged 37 to 42 years.

The participants saw both high and low contrast versions of the 30 manipulated faces, as well as 60 unmanipulated faces (“distractors”) drawn from the set used in Experiment 1 and matched in age with the 30 manipulated faces. Each participant saw three successive blocks, the first block with one version of each manipulated face (either low or high contrast), the second block with the 60 distractors and the third block with the second version of each face (either low or high contrast). The purpose of the distractors was to reduce the likelihood of participants noticing that the two presentations of each face were different. Participants were told that they could see the same person twice, but not that the images had been manipulated. The faces were randomized and counterbalanced by age within each block and by manipulation (low/high contrast) within the first and the third block. Each face was shown on the monitor until the participant typed an estimated age, between 10 and 99.

Because of the nature of our participant groups, we conducted two independent analyses to test for the effects of participant age and of participant gender. The participant age analysis tested the effects of the age of participants, the real age of the face and the type of facial contrast (low or high) on perceived age. The participant gender analysis tested the effects of participant gender, the real age of the face and the type of the facial contrast (low or high) on perceived age. The first analysis was conducted on the 60 female participants from three different age groups, and the second analysis was conducted on the 20 female and 20 male participants from the 37–42 year age group. Both analyses were performed using analysis of variance with mixed effects, as described in study 2.

A significant effect of facial contrast was found in both analyses. In the participant age analysis, faces with high contrast were perceived to be on average 1.3±0.2 years younger than those with low contrast, while in the participant gender analysis, faces with high contrast were perceived to be on average 1.1±0.2 years younger than those with low contrast (the effects of contrast were slightly different in the two analyses because the participants were mostly different). In both analyses there was no significant interaction between the age of the face and the contrast manipulation. This means that the effect of facial contrast was not different for faces of different ages.

In the participant age analysis, there was neither a significant effect of participant age nor an interaction between participant age and the effect of facial contrast manipulation. This means that there were not different effects of facial contrast manipulation in participants of different ages. In the participant gender analysis, there was a significant effect of participant gender. Male participants estimated the faces to be 1.9±0.8 years older than the female participants. It is unclear what caused this sex difference. However, there was no significant interaction between participant gender and the effect of facial contrast manipulation. This means that there were not different effects of facial contrast manipulation in participants of different genders. In summary, there were no interactions between the effects of facial contrast manipulation and the age of the face, the age of the participant, or the gender of the participant. Thus, the finding that female faces look younger with increased facial contrast is robust across different ages of faces and different ages and genders of observers.

### Studies 3a and 3b: Discussion

Although increasing facial contrast made faces look younger in both parts of Study 3, the effect was apparently large in Study 3a, but small in Study 3b. There are at least two reasons for this. One reason is that participants may have recognized the same identities across blocks in 3b (despite the intervening distractor items) and given similar responses, which would have had the effect of reducing the difference in estimated age between high and low contrast faces. The second and more fundamental reason has to do with the different procedures in the two studies. In Study 3a, the two images being compared by the participants differed only in terms of facial contrast. Wrinkles, facial sagging, age-related spots, eye and lip size, and all the other cues to aging were held constant, so that the only difference between the two faces was the degree of facial contrast. The fact that participants overwhelmingly chose the faces with higher contrast as appearing younger simply shows that facial contrast is a cue for age perception. However it does not show that facial contrast is the only cue or even the dominant cue for age perception. In Study 3b, participants estimated the age of individual faces. In this way, all static visual cues were available for perceiving the age of the face, including all the cues described above as well as any others that have yet to be discovered. Thus, the small difference in estimated age between faces with high and low contrast in Study 3b reflects the fact that there are many cues that convey facial age, and changing any single cue will not have a dramatic effect on perceived age.

This distinction can be understood in terms of task effects. Fundamentally the tasks differ in terms of whether one or many different cues are involved in the decision. In Study 3a, subjects had to decide which of two faces appeared younger, when the two faces differed only in terms of a few aspects of facial contrast. Thus, these aspects of facial contrast were the only cues available for performing the task. In this way Study 3a directly addressed the question of whether these aspects of facial contrast play *any significant* role in age perception. The answer was a resounding “yes”, as subjects chose the face with higher contrast as appearing younger in nearly every trial. In contrast, subjects in Study 3b had to decide how old a single face appeared. In this task, *all* of the facial cues to age were available for performing the task. These included all the known cues such as those described above as well as the manipulated aspects of facial contrast, and presumably many other currently unknown cues to age. Because so many different cues were available to the subjects to make their age estimation, we would not expect manipulations of any *single* cue to have a large effect on apparent age. The fact that subjects did rate the faces with greater facial contrast as appearing significantly younger is consistent with the idea that these manipulated aspects of facial contrast are cues for age perception. Some other studies investigating facial cues to age have used methods similar to study 3b and so by comparing the present results with those of these other studies we can investigate the relative importance of facial contrast for age perception.

Given that there are many different cues to facial age that are possible to use when estimating the age of a face, a remaining question is how the results of Study 3b compare with results of other studies manipulating different age-related cues. In Study 3b, faces with high contrast were perceived to be on average 1.3±0.2 (participant age analysis) or 1.1±0.2 (participant gender analysis) years younger than faces with low contrast. In one recent study images of Caucasian faces aged in their 60s were perceived to be 3.6 years younger after digital removal of wrinkles [34]. Though the methods and stimuli are not directly comparable for a variety of reasons, the effect of manipulating facial contrast in the present work was about one third the size of the effect of entirely removing wrinkles in the other study. Yet another study investigated actual surgical procedures and found that apparent age is reduced by 2.5 years after a laser resurfacing procedure (i.e., treatment of wrinkles, solar lentigines, sun damage, scars) and by 4.6 years after a complete facelift (i.e., surgical removal of both wrinkles and tissue slackening) for patients in their 40s and older [35]. Although these two cosmetic surgery procedures result in dramatic changes in facial appearance, the difference in estimated age is still on the order of only a few years. Thus, even major manipulation of several different age-related cues does not dramatically change the apparent age of the face. Nonetheless, the effects of these procedures are larger than that found for facial contrast manipulation in Study 3b, which suggests that facial contrast is a weaker cue for age perception than are wrinkles and sagging.

In summary, the results from the two parts of Study 3 taken together support the notion that facial contrast plays a significant and meaningful role in age perception. However, the role played by facial contrast is smaller than that played by well-known age-related cues such as wrinkling and sagging.

## General Discussion

Several aspects of facial contrast – the luminance and color differences between the facial features and the skin surrounding those features – were found to decrease with age. These included the luminance contrast around the eyes and eyebrows, the red-green (a*) contrast around the mouth and eyes, and the yellow-blue (b*) contrast around the eyes. These same attributes of facial contrast were negatively correlated with perceived age. Yellow-blue (b*) contrast of the mouth increased with age and was positively correlated with perceived age. Finally, manipulations of facial contrast changed the apparent age of the face. In two studies, faces with increased facial contrast were judged to be younger than those with decreased contrast. Collectively these results demonstrate that facial contrast is a visual cue that changes with age, and is used by observers in perceiving the age of a face.

Facial contrast is known to be a cue for sex classification and facial attractiveness judgments [22]–[26]. Here we extended the definition of facial contrast to include color contrast and contrast around the eyebrows, and have shown that facial contrast is also a cue for age perception. Facial contrast can be added to the list of cues that are involved in age perception, including wrinkles, sagging, size of the eyes and lips, and uniformity of skin reflectance, to name a few [21]. However, it should be noted that the L* contrast around the mouth did not change with age, and we have no evidence that it is related to the perception of age. Thus, the aspects of facial contrast that change with age are not exactly the same as those that differ between male and female faces.

Bruce and Young [36] proposed that there is a link between the perception of age and the perception of femininity (p. 118). Our findings here support this notion, by showing that a sexually dimorphic feature–facial contrast–also changes with age. Higher facial contrast is typical of female faces but also of younger faces. This suggests the possibility that there is a decrease in the apparent femininity of female faces as they age. A larger positive effect of cosmetics on physical attractiveness has been observed in older women compared to younger women [37]. It may be that increases in facial contrast caused by cosmetics have a larger effect on apparent femininity in older faces than in younger faces.

While contrast decreases with age, the application of cosmetics serves to increase the luminance portion of facial contrast [25]. The effect of cosmetics on the color portion of facial contrast has not been investigated. The application of lipstick not only darkens the lips but should also increase red-green contrast of the lips, which we found to be higher in younger faces. Women also apply eye make-up (i.e., mascara, eye-liner and eye shadows) in a way that increases luminance and color contrast of the eyes, as well as eyebrow make-up (e.g., color pencils or permanent make-up) that increases luminance contrast of this region. Cosmetics are known to increase the apparent attractiveness [37]–[42], femininity [39], [43], and healthiness [43], [44] of female faces. Elsewhere we have suggested that by increasing facial contrast, cosmetics increase facial femininity and hence attractiveness of the female face [25], [45]. Here we add to this account with the suggestion that another function of cosmetics is to make the face look younger by increasing facial contrast.

One of the most important components of cosmetic use is the application of lipstick. Red shades are commonly used for lipstick, which should have the effect of darkening lips that are naturally pinkish. Indeed, typical application of cosmetics have been shown to make the lips darker and to increase the luminance contrast around the lips, making the female face more sex-typical [25]. However, red lipstick should also have the effect of increasing red-green contrast. Stephen & McKeegan [26] found that people increase the redness (a*) of the lips to make a female face appear more feminine and attractive. Elliot & Niesta [46] found that pictures of women are perceived by men as more attractive and sexually desirable when they are associated with the color red–whether by the placement of a red border around the picture, or the presence of a red colored shirt on the woman. They offered this association as a possible reason for the common use of red lipstick. Here we found that the red-green contrast around the lips decreases with age. Because of this, we propose that red lips are associated with youthfulness as well as with femininity and sexuality.

As this is the first investigation of the role of facial contrast in age perception, many questions remain. One group of questions involves the relative importance of the different aspects of facial contrast in the perception of age. Are certain features or color dimensions more important than others for age perception? And do these different aspects of facial contrast have independent or interdependent effects on age perception? These questions cannot be directly answered by the current work because we manipulated all the age-related contrast features at once. However, a reasonable assumption is that the feature changes that are more strongly related to age (e.g. luminance contrast around the eyebrow or redness contrast around the lips) play a larger role in the perception of age than those feature changes that were more weakly related to age (e.g. luminance contrast around the eyes or yellowness contrast around the lips). A similar issue is that we did not vary the magnitude of the manipulations, and so questions about how much of a change is required for an effect on age perception, or whether more of a change is required to have an effect on different faces will require further research.

Another group of open questions about the role of facial contrast in the perception of age relates to the kinds of faces for which it is a useful cue. Here we investigated only Caucasian females between 20 and 69 years of age, so we cannot directly address the question of whether facial contrast is a cue for perceiving the age of people outside of this demographic group. Because we only investigated faces from 20–69 years of age in studies 1 and 2, and faces from 23–59 years of age in study 3, additional work will be needed to determine whether facial contrast is a cue for age perception in faces of children or the elderly. We suspect that facial contrast is also a cue for age perception in adult Caucasian male faces because facial aging processes are largely similar in male and female faces [47], and because skin color has been shown to be a cue to age perception in both female [17] and male faces [48]. Further, the grayscale morphed averages of Caucasian male faces of different ages shown in Figure 1 of Burt & Perrett’s 1995 study [13] appear to show decreases in facial contrast with age. The ways that faces of different races change with age are believed to be largely similar [49]–[51], which suggests the likelihood that facial contrast is a cue to age not only for Caucasian faces but also for faces of racial groups. However, even if facial contrast does decrease with age in faces of other races, including male faces, the particular aspects of facial contrast that vary with age and are used as cues for age perception may not be exactly the same in different racial groups or between the sexes, further emphasizing the need to investigate these other demographic groups.

### Conclusions

We have shown that aspects of facial contrast decrease with age in adult Caucasian female faces, and that facial contrast is associated with perceived age. Faces with lower facial contrast look older than faces with higher facial contrast, and the same face can be made to appear younger or older by increasing or decreasing facial contrast. Thus, facial contrast can be added to the list of known signs of aging. Among the signs of aging, facial contrast is of particular interest because of its clear relationship to cosmetics use.
